# Monitoring the Presence of 13 Active Compounds in Surface Water Collected from Rural Areas in Northwestern Spain

**DOI:** 10.3390/ijerph110505251

**Published:** 2014-05-15

**Authors:** Alejandra Iglesias, Carolina Nebot, Beatriz I. Vázquez, Claudia Coronel-Olivares, Carlos M. Franco Abuín, Alberto Cepeda

**Affiliations:** 1 Department of Analytical Chemistry, Nutrition and Bromatology, Faculty of Veterinary Medicine, University of Santiago de Compostela, Lugo 27002, Spain; E-Mails: alejandra.iglesias@usc.es (A.I.); beatriz.vazquez@usc.es (B.I.V.); carlos.franco@usc.es (C.M.F.A.); alberto.cepeda@usc.es (A.C.); 2 Instituto de Ciencias Básicas e Ingeniería, Área Académica de Química, Universidad Autónoma del Estado de Hidalgo, Carretera Pachuca-Tulancingo Km. 4.5, Ciudad del Conocimiento, Mineral de la Reforma, Hidalgo 42184, Mexico; E-Mail: ccoronel@uaeh.edu.mx

**Keywords:** drugs, urban, rural, LC-MS/MS, environmental water, Spain

## Abstract

Drug residues are considered environmental contaminants, and their occurrence has recently become a matter of concern. Analytical methods and monitoring systems are therefore required to control the continuous input of these drug residues into the environment. This article presents a suitable HPLC-ESI-MS/MS method for the simultaneous extraction, detection and quantification of residues of 13 drugs (antimicrobials, glucocorticosteroids, anti-inflammatories, anti-hypertensives, anti-cancer drugs and triphenylmethane dyes) in surface water. A monitoring study with 549 water samples was carried out in northwestern Spain to detect the presence of drug residues over two sampling periods during 2010, 2011 and 2012. Samples were collected from rural areas with and without farming activity and from urban areas. The 13 analytes were detected, and 18% of the samples collected showed positive results for the presence of at least one analyte. More collection sites were located in rural areas than in urban areas. However, more positive samples with higher concentrations and a larger number of analytes were detected in samples collected from sites located after the discharge of a WWTP. Results indicated that the WWTPs seems to act as a concentration point. Positive samples were also detected at a site located near a drinking water treatment plant.

## 1. Introduction

Around the world, drugs consumption in both human and veterinary medicine has been increasing year after year. The consumption of pharmaceuticals in the EU is substantial, with approximately 3,000 different active substances such as analgesics and anti-inflammatory drugs, contraceptives, antibiotics, beta-blockers, lipid regulators and neuroactive compounds being commonly used in human medicine [1]. These compounds enter the environment through many routes, including manufacturing, formulation, distribution, use and disposal. The use of pharmaceuticals by individuals is the main route. After ingestion, metabolites or drugs that are not completely metabolised in the body are expelled in faeces or urine and reach drains. Wastewater treatment plants (WWTPs) have no specific technologies to eliminate drugs entirely from the waste stream, so active compounds (drugs and metabolites) enter the aquatic environment straightforward from the effluents are discharged daily into rivers, making these residues pseudo-persistent pollutants. Pharmaceuticals widely used in the treatment of animals such as the antimicrobials, sulfonamides and quinolones can also be employed in the treatment of human infections. These drugs could be released directly to the environment through the extensive livestock-raising operations and can accumulate in manure pits or livestock waste that may be used to fertilise agricultural lands [2]. The result of these practices is that any residues of drug administered reach via runoff, rivers, lakes and seas and via filtration, groundwater, aquifers and wells. The direct contamination of aquatic environments by aquaculture can lead to the exposure of aquatic and sediment dwelling organisms to the contaminants [3]. The triphenylmethane dyes, malachite green (MG) and brilliant green (BG), originally used as dyeing agents in the textile and paper industries, are also observed as contaminants of the aquatic environment. The dyes were introduced illegally in 1933 as ecto-parasiticides, fungicides and antiseptics in aquaculture because of their broad fungicidal and antiparasitical activity, and the dyes are effective against Gram-positive microorganisms [4]. However, MG and BG are toxic to multiple organs in mammals and have negative effects on the immune and reproductive systems as well as genotoxic and carcinogenic properties [5,6], so their use in aquaculture is forbidden.

The reason for the development of interest in the research is that reports on the occurrence of pharmaceuticals in the aquatic environment show that the pharmaceuticals are ubiquitous. The continued input of these compounds into the environment, even at low concentrations, potentially serves to sustain chronic exposure for aquatic organisms [7]. Toxic effects in non-target organisms have previously been reported by Jung *et al.*, Kim *et al.*, Li *et al.* and Madureira *et al.* [8,9,10,11]. Sulfonamides, propanolol, trimethoprim and diclofenac, *inter alia*, have been demonstrated to exert toxicity on plants, soil organisms, freshwater crustaceans, fish and zebrafish.

Pharmaceutical pollutants are being discharged daily and continuously, albeit their detection in the aquatic environment can be complicated because these compounds are typically present at levels of ng·L^−1^ and pg·L^−1^ [7,12]. Detection of pharmaceuticals is influenced by the different properties of the multiple classes of drugs. Specific and suitable methods therefore need to be developed to identify and quantify the drugs in the aquatic environment. The analytical methods employed for the analysis of drugs are also varied, and the sample preparation procedure generally involves the use of large sample volumes, multiple extractions, sophisticated sample clean up, and/or derivatisation prior to analysis. Following extraction, depending on the nature of the target compound, pharmaceuticals are identified and quantified using a variety of analytical instruments. Analytical techniques including high performance liquid chromatography (HPLC) and gas chromatography (GC) allow the analysis of multiple analytes in the same extract when coupled to a mass spectrometer (MS), the combination of these techniques is commonly employed in environmental analyses [13,14,15,16,17,18,19]. However, detectors other than MS such as ultraviolet (UV), fluorescence (FL), chemiluminescence (CL) and other techniques such as capillary electrophoresis (CE) are also employed [20,21,22,23,24,25].

In the Mediterranean area of Spain, pharmaceuticals such as antimicrobials and anti-inflammatories and other compounds such as drugs of abuse have been reported several times in the 21st century [26,27,28,29,30,31]. The presence of pharmaceuticals has also been reported in Sevilla, Madrid and Galicia [32,33,34] surface waters. However, none of the publications cited, to the author’s knowledge, have previously investigated the presence of antimicrobials, anti-hypertensives, anti-cancer drugs, corticosteroids, anti-inflammatories and triphenylmethane dyes in river water samples, simultaneously. The geographical area investigated in this work contains the most important river in the Galician area, running through areas with high population density as well as areas dedicated to agriculture and livestock, thus indicating the importance of this community in the production of food from animal and vegetable origins. Because there are trout farms and paper industries in the sampling area, the possibility of finding residues of MG and BG in the aquatic environment must be considered.

The aim of this work is to present a method using HPLC-MS/MS for the simultaneous identification and quantification of 13 target compounds (seven antimicrobials, one anti-hypertensive, one anti-cancer drug, one synthetic corticosteroid, one non steroidal anti-inflammatory and two triphenylmethane dyes) in surface water. In particular, diclofenac, sulfamethoxazole, trimethoprim and oxolinic acid were included in the study because they are drugs commonly used in human medicine and their presence in environmental waters has been reported by different authors [14,17,32].

The assessment of the level of pollution generated in the production of food from animal origin derived from the use of the selected drugs has been carried out by analyzing water samples collected from rural areas with high and low density of farming activities. Urban areas located close to the discharge of WWTPs, were included in the study for comparison and based on the common presence of some of the drugs in these types of sampling points. Therefore, the method was validated in house and has been applied to the analysis of 549 surface water samples collected in rural and urban areas of the Galician environment during two different sampling periods.

## 2. Experimental Section

### 2.1. Chemicals, Reagents and Stock Solutions

Brilliant green, diclofenac, difloxacin, enrofloxacin, malachite green, marbofloxacin, oxolinic acid, propanolol, sarafloxacin, sulfamethoxazole, tamoxifen, triamcinolone and trimethoprim (all purity > 98%) and the internal standards (ISs) sulfadoxine-d_3_ and malachite green-d_5_ picrate were purchased from Sigma-Aldrich (St. Louis, MO, USA). All the therapeutic and chemical properties of the selected drugs are presented in Table 1. Methanol (HPLC-grade, ≥99.9%) was obtained from Scharlau Chemie (Barcelona, Spain), and formic acid (purity > 99% for analysis) was purchased from Acros Organics (Geel, Belgium). Hydrochloric acid solution (1 N HCl) was purchased from Merck (Darmstadt, Germany). Purified water was prepared in house with a Milli-Q water system from Millipore (Bedford, MA, USA), and nitrogen gas (purity > 99.98%) was generated by an in-house nitrogen generator from Peak Scientific Instruments Ltd. (Chicago, IL, USA).

**Table 1:** Therapeutic class and chemical properties of the selected drugs.

Analyte	Therapeutic Class	CAS Number	Formula
Brilliant Green	Triphenylmethane dye	633-03-4	C_27_H_34_N_2_O_4_S
Diclofenac	Anti-inflammatory	15307-79-6	C_14_H_11_Cl_2_NO_2_
Difloxacin	Antimicrobial	98106-17-3	C_21_H_19_F_2_N_3_O_3_
Enrofloxacin	Antimicrobial	93106-60-6	C_19_H_22_FN_3_O_3_
Malachite Green	Triphenylmethane dye	2437-29-8	C_23_H_25_ClN_2_
Marbofloxacin	Antimicrobial	115550-35-1	C_17_H_19_FN_4_O_4_
Oxolinic Acid	Antimicrobial	14698-29-4	C_13_H_11_NO_5_
Propanolol	Anti-hypertensive	525-66-6	C_16_H_21_NO_2_
Sarafloxacin	Antimicrobial	98105-99-8	C_20_H_17_F_2_N_3_O_3_
Sulfamethoxazole	Antimicrobial	723-46-6	C_10_H_11_N_3_0_3_S
Tamoxifen	Anti-cancer	10540-29-1	C _26_ H _29_ NO
Triamcinolone	Glucocorticoid	124-94-7	C_21_H_27_FO_6_
Trimethoprim	Antimicrobial	738-70-5	C_14_H_18_N_4_O_3_

The drugs were accurately weighed (±0.0001 g) on an analytical balance from Ohaus^®^ GA200 (Nänikon, Switzerland) to prepare stock solutions of individual compounds at a concentration of 0.6 mg·mL^−1^ in methanol. These stock solutions were mixed with 0.1% of formic acid in methanol to obtain a stock solution of 1 µg·mL^−1^, and this solution was further diluted with 0.1% of formic acid in methanol to obtain standard mixtures of drugs at 12.5, 25, 50, 75, 100 and 150 ng·mL^−1^. The stock solutions of the ISs (sulfadoxine-d_3_ and malachite green-d_5_ picrate) at 0.6 mg·mL^−1^ were mixed to obtain a working stock solution of 1 µg·mL^−1^ with 0.1% formic acid in methanol. All of the standard solutions were stored in the dark at −18 °C for a maximum of six months.

### 2.2. Equipment

Samples were analysed on an HPLC-MS/MS system consisting of an HPLC model 1200 G1312A from Agilent Technologies (Waldbronn, Germany) with a binary pump, a degasser and an autosampler. The MS was a model API 4000™ from Applied Biosystems MSD Sciex Instruments (Toronto, ON, Canada) with an integrated TurboIonSpray^®^ for molecule ionisation. The software Analyst 1.4.1, also from Applied Biosystems, MSD Sciex, was employed to acquire the data and control the system.

The chromatographic analyses were performed by injecting 10 µL of the extract into a Synergi 2.5 µm Polar-RP 100A column (50 × 2.0 mm) connected to a Polar-RP security-guard cartridge (4.0 × 2.0 mm), which were both obtained from Phenomenex (Macclesfield, UK). An MS2 Minishaker vortex mixer from IKA^®^ (Staufen, Germany) and a vacuum station manifold with Strata^®^-X solid phase extraction (SPE) cartridges (60 mg, 3 mL), which were both obtained from Phenomenex (Macclesfield, UK), and a TurboVap^®^ II evaporator from Zyrmark (Hopkinton, MA, USA) were employed for sample preparation and extraction.

The physical and chemical parameters (nitrites, ammonium, conductivity, turbidity and pH) were measured for each collected sample. These analyses utilised the following equipment and kits: Visocolor^®^ ECO nitrite test (0.02–0.5 mg·L^−1^ NO_2_^−^) and ammonium 3 (0.2–3 mg·L^−1^ NH^4+^), both from Macherey-Nagel GmbH & Co. KG (Düren, Germany); a conductivity meter model CON6/TDS6 (Hand-held Conductivity/TDS Meter) from Eutech Instruments Pte. Ltd./Oakton Instruments (Illinois, IL, USA); a turbidimeter model TN-100 from Eutech Instruments Pte. Ltd. (Singapore, Singapore); and a pH meter model MicropH 2000 from Crison (Barcelona, Spain).

### 2.3. HPLC-MS/MS Conditions

Separation of analytes was achieved using a gradient mixture of two components, A (0.1% formic acid in water) and B (0.1% formic acid in methanol). The gradient programme employed is shown in Table 2, and the flow rate was 0.2 mL·min^−1^ during the whole run.

**Table 2:** Gradient program of the HPLC-MS/MS method developed.

Total Time (min)	Mobile Phase A (%)	Mobile Phase B (%)
0	90	10
2	90	10
7	50	50
15	25	75
24	0	100
28	0	100
33	75	25
36	90	10
40	90	10

Notes: A: 0.1% formic acid in water; B: 0.1% formic acid in methanol.

Mass spectrometric measurements were performed using positive electrospray ionisation (ESI^+^), and drug identification was performed using two multiple reaction monitoring (MRM) transitions and their retention times (t_R_). Standard solutions of the individual drugs in a concentration of 1 µg·mL^−1^ in 0.1% formic acid in methanol were infused directly into the mass spectrometer, using a 1 mL syringe pump (model Gastight^®^ 1001), which was purchased from Hamilton (Bonaduz, Switzerland), at a flow rate of 5 µL·min^−1^. The optimum cone voltage and collision gas energy were selected when they gave the most intense signals. Then, the HPLC with a Synergy-Polar column was connected to the MS.

To monitor a specific transition between a precursor and a product ion, the following MS parameters needed to be set: declustering potential (DP), entrance potential (EP), collision energy (CE) and cell exit potential (CXP). These parameters varied for each compound and changed automatically during the run, as summarised in Table 3. The following MS parameters were kept constant during the run: a source temperature of 650 °C, a vacuum gauge of 4.1·10^−5^ Torr, an ion spray voltage of 5,000 V and a curtain gas pressure of 12 psi. The first ion source was set at 45 psi, and the second ion source was set at 50 psi. For all monitored transitions, the dwell time was 10 ms.

**Table 3:** Retention time (t_R_), precursor and product ions and MS parameters employed to identify the selected drugs.

Analyte	t_R_ (min)	Precursor (*m*/*z*)	Product 1 (*m*/*z*)	Product 2 (*m*/*z*)	Precursor > Product Ion 1			
					DP	EP	CE	CXP
**Brilliant Green**	34.09	385	341	241	61	10	53	10
**Diclofenac**	20.29	296	214	250	51	10	43	14
**Difloxacin**	15.44	400	299	356	71	10	27	32
**Enrofloxacin**	14.91	360	316	342	76	10	28	8
**Malachite Green**	25.12	329	208	165	111	10	43	18
**Marbofloxacin**	13.74	363	72	320	76	10	51	4
**Oxolinic Acid**	16.62	262	216	160	36	10	39	14
**Propanolol**	15.97	260	116	183	106	10	23	10
**Sarafloxacin**	16.05	386	342	299	76	10	23	22
**Sulfamethoxazole**	13.20	254	156	92	81	10	25	14
**Tamoxifen**	21.2	372	340	235	106	10	73	14
**Triamcinolone**	15.34	395	375	357	66	10	15	6
**Trimethoprim**	12.05	291	230	123	56	10	35	20
**Sulfadoxine d_3_**	13.70	314	156	108	41	10	27	12
**Malachite green d_5_ picrate**	25.8	334	213	170	126	10	47	24

Notes: DP: Declustering potential; EP: Entrance potential; CE: collision energy; CXP: cell exit potential.

### 2.4. Study Site Description

The geographical area investigated is located in the extreme northwest (NW) portion of the Iberian Peninsula, just north of Portugal. The Miño River is an international river with a length of approximately 340 km. The source of the Miño River lies in Pedregal of Irimia, which is 50 km north of Lugo in Galicia, Spain (Serra de Meira, Lugo, at 750 m elevation) and in the last 75 km, the river defines the border between Portugal and Spain. The Miño River is the most important drainage basin in NW Spain, extending over 17,757 km^2^ with abundant areas dedicated to agriculture and livestock distributed along its path to the Atlantic Ocean [35]. The landscape of Galicia is dominated by the valleys created by the Miño River and its tributaries. Samples were collected from the Miño River and one of its main tributaries, the Asma River. The study area encompassed the upper basin, which includes the metropolitan area of Lugo, with approximately 98,000 residents (INE 2011) and areas dedicated to agriculture and farm production.

### 2.5. Sampling Strategy, Method and Conservation

**Figure 1:**
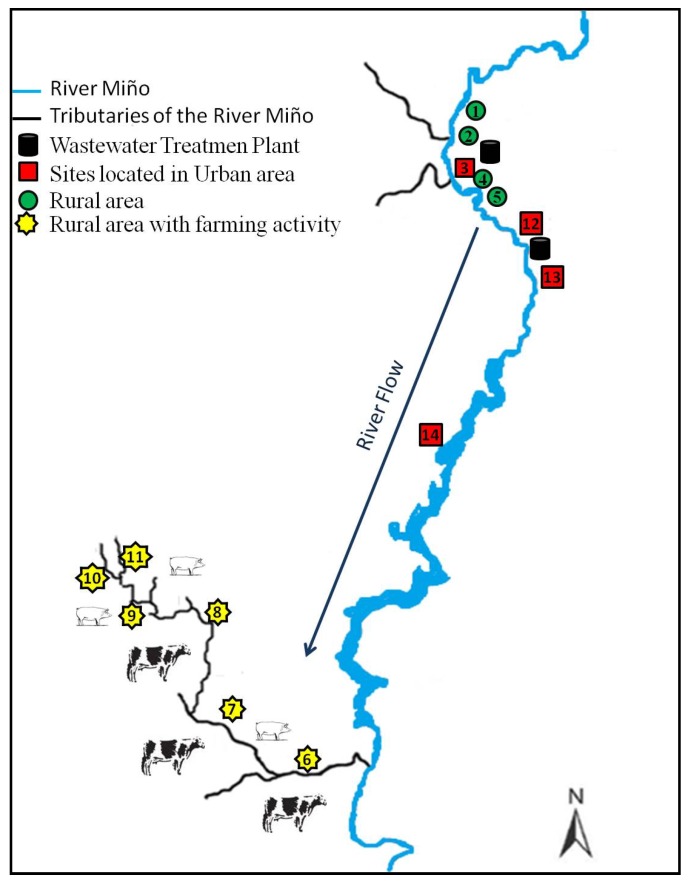
Locations of the sampling sites selected for the monitoring study.

Our sampling strategy was designed based on previous work where veterinary drugs were investigated in river water from different areas with and without farming activity using a total of 14 sampling points. Six of the points were located in rural areas and the other eight were chosen at different points along the Miño River, Galicia’s largest river, which goes across both rural and urban areas (Figure 1).

The method was developed and validated with river water samples collected from the Miño River, and the monitoring study was conducted with Galician fresh water. A total of 267 river samples of 1-L were collected in 1-L polyethylene plastic vessels during two different sampling periods, the first sampling period over a nine-week period from October to December 2010 and the second sampling period over a seven-month period between November 2011 and May 2012. The sampling sites were distributed along the Miño River between the villages of Rábade and Chantada in the province of Lugo. Of a total of 14 points, eight were located at the Miño River, one in its tributary the Asma River and five in streams and brooks (Figure 1).

All the samples for method validation and monitoring studies were filtered upon arrival at the laboratory and divided into replicate samples of 500 mL and were stored at 4 °C until analysis was carried out, which was always performed the day after sampling. The extracts were stored at −18 °C until their HPLC-MS/MS analysis was performed which took place within no more than seven days after the extraction. To quantify the analytes correctly all the river water samples were spiked with 50 µL of the IS solution and a blank and two control samples (Milli-Q water and Milli-Q water samples spiked at 75 and 150 ng/L, respectively) were filtered, stored and processed in the same way as our natural water samples for identification of contamination risk during sample processing.

### 2.6. Analytical Procedure

Analytical determinations were carried out using a modification of a previously reported method [36] based on solid-phase extraction (SPE) and determination by high performance liquid chromatography coupled to tandem mass spectrometry. Filtered samples were acidified with 1 N HCl solution to a pH of 3 and loaded in to a SPE cartridge (Strata^®^-X, Phenomenex, Torrance, CA, USA) activated with 4 mL of methanol and 4 mL of water at pH 3. The analytes were eluted with 8 mL of methanol and evaporated to dryness under a nitrogen stream at 45 °C. The extracts were reconstituted with 200 µL of 0.1% formic acid in methanol and stored at −18 °C until further analysis by HPLC-MS/MS. The extracts were analysed within one week following extraction.

The samples employed for the determination of physical and chemical parameters (nitrites, ammonium, conductivity, turbidity and pH) were stored at 4 °C in the laboratory until analysis, which was carried out within one day of sampling. The physical and chemical parameters were determined following the manufacturers’ instructions for the respective kits and equipment.

### 2.7. Validation Procedure

As the method employed is a modification of a previously reported method, validation had to be performed.

The linear response of the instrument to standard solutions containing all the selected drugs at six different concentrations (1, 5, 10, 50, 100 and 200 ng·mL^−1^) was investigated. These standard solutions were employed to obtain the instrument calibration curves (ICCs). The instrument detection limit (IDL) was defined as the concentration that gave a signal-to-noise (S/N) ratio above 3, and the instrument quantification limit (IQL) was defined as the concentration that gave an S/N above 10. The linearity of the whole method, extraction and HPLC-MS/MS detection, was evaluated using matrix-matched samples with river water samples spiked with all the selected drugs at the following concentrations: 1, 5, 10, 20, 50, 75, 100, 150 and 200 ng·L^−1^. These samples were employed to build sample calibration curves (SCCs) and to determine the limit of detection (LOD) for the method, which is defined as the lowest concentration that gave an S/N above 3 and a method limit of quantification (LOQ) at an S/N above 10. Once the LOD was established, the method detection limit (MDL) was also calculated following the Environmental Protection Agency (EPA) procedure from CFR 136, Appendix B criteria [37]. The MDL is defined as the minimum concentration of a substance that can be measured and reported with a 99% confidence that the analyte concentration is greater than zero. The MDL is determined from the replicate analysis of a sample in a given matrix containing the analyte.

The analytical method was validated in terms of selectivity, linearity, LOD, LOQ, MDL repeatability, precision and accuracy.

### 2.8. Statistical Analysis

Once the results from the identification and quantification of the selected analytes and the results from the determination of the physicochemical parameters were obtained from the monitoring study, they were analysed using the software PASW Statistics 18 (SPSS Inc., Chicago, IL, USA) to identify statistically significant trends in the drug concentrations. The effects of the weather conditions, sampling site characteristics (rural and urban areas), sampling date and physical and chemical parameters (nitrites, ammonium, conductivity, turbidity and pH) of the water samples collected were tested using one-way ANOVA (*p* < 0.05).

## 3. Results and Discussion

### 3.1. Optimisation of the HPLC-MS/MS and Extraction Protocols

Initially, the method employed by Nebot *et al.* [36] was selected for the analysis of the collected samples. However, based on the capability of a Synergy-Polar column in the laboratory, this column was selected to perform the analysis instead of a Luna column. This change of the chromatographic columns could explain the results obtained for the paracetamol peak, which did not have a Gaussian shape. The results of the analysis of this compound consequently had to be discarded. More compounds were also included in the method (brilliant green, cefalexin, difloxacin, enrofloxacin, gentian violet, leucomalachite green, malachite green, marbofloxacin, norfloxacin, oxolinic acid, sarafloxacin and triamcinolone). The mobile phase employed was different: methanol and water acidified with formic acid instead of ammonium acetate was employed because the best peak resolution and peak shape were achieved with a combination of these solvents. Formic acid aided in the prevention of peak tailing and provided sufficient ionisation [38,39]. The reproducibility of the t_R_ of the selected drugs was evaluated through the RSDs, and their values were in the range between 0.44% and 5.40%.

The SPE protocol described by Nebot *et al.* [36] was initially employed but for the final method some modification were applied. The cartridges were conditioned with 1 mL less of methanol and water, the sample flow was the same but the elution of the drugs was conducted differently. Methanol alone was employed instead of acetone and methanol because a larger number of analytes were recovered. Instead of 2 L of sample, a volume of 500 mL was employed to spend as little time as possible during extraction and to avoid product degradation due to the laboratory conditions. The acidification of the water samples to pH 3 with 1 N HCl solution was performed to improve the interaction between the elution solvent and the analytes in the SPE [40]. From 19 pharmaceuticals (brilliant green, cefalexin, diclofenac, difloxacin, enrofloxacin, erythromycin, gentian violet, leucomalachite green, malachite green, marbofloxacin, mefenamic acid, norfloxacin, oxolinic acid, propanolol, sarafloxacin, sulfamethoxazole, tamoxifen, triamcinolone and trimethoprim), a total of 13 analytes were extracted satisfactorily. Recoveries of cefalexin, erythromycin, gentian violet, leucomalachite green, mefenamic acid and norfloxacin could not be taken into account because of their low recoveries. Some steps during the extraction of the fortified samples led to degradation and low recoveries of the analytes. In particular, the pH adjustment inhibited the extraction of erythromycin and mefenamic acid. Recoveries of trimethoprim, sulfamethoxazole, propanolol, diclofenac and tamoxifen from fortified river water were higher in this study compared to Nebot *et al.* [36] most likely due to a different extraction protocol, but the LOD and LOQ were lower certainly due to lower sample volume. Another factor that could have improved recoveries would be the use of an internal standard, contrasting with the procedure of Nebot *et al.* [36], who quantified 12 human drugs without the employment of any IS. In this research, the IS sulfadoxine-d_3_ was employed to quantify the selected drugs in accordance with Hao *et al.*, Hilton *et al.* and Kasprzyk-Hordern *et al.* [41,42,43], who employed one or two ISs to quantify 13 drugs from different therapeutic classes in water samples.

A test was conducted to determine the percent of compound lost during the extraction protocol, in which a battery of water samples was fortified at a concentration of 25 ng·L^−1^ after the evaporation step. The results showed a loss between 40%–50% of diclofenac, MG, oxolinic acid, propanolol, sulfamethoxazole and tamoxifen during the extraction step.

### 3.2. Method Validation

The selectivity of the method was investigated by comparing the two MRM transitions of the selected analytes obtained from a standard solution, non-fortified river samples and fortified river samples. The method selectivity was demonstrated by noting the absence of interference peaks at the t_R_ of the analytes and IS for both selected transitions.

When a standard solution containing a mixture of the analytes was injected at different concentrations, the IDL obtained ranged from 0.2 to 6.4 ng·mL^−1^, and the IQL ranged from 0.3 to 6.7 ng·mL^−1^ (Table 4). A linear response was also observed for concentrations between 1–200 ng·mL^−1^, with a mean *R*^2^ higher than 0.991. The linearity of the whole procedure, samples spiked and extracted as described previously, was observed to have a mean *R*^2^ above 0.979 for a concentration range between 1 and 200 ng·L^−1^ (Table 5).

For fortified samples, the LOD was in the range of 1–3.43 ng·L^−1^, and the LOQ was in the range of 3.0–5.15 ng·L^−1^ (Table 4). Although there are reports of lower LODs and LOQs, for example, for tamoxifen (0.03 and 0.08 g·L^−1^) [36], triamcinolone (0.5 and 1.67 ng·L^−1^) [44], and marbofloxacin (LOD = 0.8 ng·L^−1^) [40], other studies present LOD and LOQ values for enrofloxacin (LOD = 34 ng·L^−1^, LOQ = 120 ng·L^−1^) and trimethoprim (LOD = 91 ng·L^−1^, LOQ = 310 ng·L^−1^) higher than those reported in this work [45] where ion trap MS was used by Dinh *et al.* [14] for enrofloxacin (LOD = 3.3 ng·L^−1^, LOQ = 11 ng·L^−1^) and oxolinic acid (LOD = 1.7 ng·L^−1^, LOQ = 5.7 ng·L^−1^) and sarafloxacin (LOD = 1.1 ng·L^−1^, LOQ = 3.6 ng·L^−1^). Martín *et al.* and Batt *et al.* reported LOD and LOQ values for propanolol (LOD = 1 ng·L^−1^, LOQ = 2 ng·L^−1^ and LOD = 0.6 ng·L^−1^, LOQ = 2.1 ng·L^−1^) [32,46], lower than the present work. However, for the compounds diclofenac, sulfamethoxazole and trimethoprim (LOD = 15 ng·L^−1^, LOQ = 49 ng·L^−1^, LOD = 8 ng·L^−1^, LOQ = 28 ng·L^−1^, LOD = 6 ng·L^−1^, LOQ = 20 ng·L^−1^), the values were considerably greater [32]. Data from Ashton *et al.* [47] showed much higher values than those obtained in this work for sulfamethoxazole, trimethoprim, propanolol, diclofenac and tamoxifen. To the authors’ knowledge, no LOD and LOQ values are available for malachite green and brilliant green in surface water to compare with those obtained in this study.

The MDL was calculated for each analyte using river samples fortified with the pharmaceuticals at a concentration of 25 ng·L^−1^. MDL values for each analyte are summarised in Table 4, and the range was between 2 and 15.1 ng·L^−1^. The MDL values for diclofenac and sulfamethoxazol achieved during this research were lower than those published by Cahill *et al.* and Yang *et al.* [48,49]; Muñóz *et al.* reported the same MDL for propanolol (2 ng·L^−1^) [50]. However, data obtained for trimethoprim and enrofloxacin were higher than those reported by Senta *et al.*, who achieved MDLs for these compounds between 1–8.5 ng·L^−1^ [39]. Batt *et al.* also report MDLs for sulfamethoxazole and propanolol (5.5 and 1.3 ng·L^−1^) [46], and Kasprzyk-Hordern *et al.* report an MDL for diclofenac (0.05 ng·L^−1^) [51]. Once again, no data for malachite green and brilliant green in surface water samples were found.

**Table 4:** Instrument detection limit (IDL), instrument quantification limit (IQL), limit of detection (LOD), limit of quantification (LOQ) and method detection limit (MDL).

Analyte	IDL (ng·mL^−1^)	IQL (ng·mL^−1^)	LOD (ng·L^−1^)	LOQ (ng·L^−1^)	MDL (ng·L^−1^)
**Brilliant Green**	0.2	0.3	2.6	3.0	7.4
**Diclofenac**	0.7	0.8	2.0	2.10	11.1
**Difloxacin**	0.3	0.5	1.0	3.0	8.8
**Enrofloxacin**	5.4	5.8	1.37	5.15	8.1
**Malachite Green**	0.2	0.3	1.5	3.3	10.9
**Marbofloxacin**	0.2	0.3	1.0	3.0	8.1
**Oxolinic Acid**	6.4	6.7	3.43	3.86	9.3
**Propanolol**	0.2	0.3	1.0	3.0	2.0
**Sarafloxacin**	4.6	4.8	2.1	5.2	11.9
**Sulfamethoxazole**	0.2	0.3	1.0	3.0	11.2
**Tamoxifen**	0.2	0.3	2.0	3.0	13.5
**Triamcinolone**	0.9	1.1	1.0	3.0	7.6
**Trimethoprim**	0.2	0.3	1.0	3.0	15.1

**Table 5:** Regression coefficients (*R*^2^), mean recoveries and relative standard deviation (RSD) of the selected veterinary drugs.

Analyte	ICC R^2^	SCC R^2^	Mean Recovery (%)	RSD (%)
**Brilliant Green**	0.996	0.989	80	20
**Diclofenac**	0.999	0.999	77	16
**Difloxacin**	0.993	0.995	60	26
**Enrofloxacin**	0.997	0.979	60	27
**Malachite Green**	0.999	0.997	79	25
**Marbofloxacin**	0.991	0.986	60	23
**Oxolinic Acid**	0.994	0.991	62	25
**Propanolol**	0.999	0.999	93	10
**Sarafloxacin**	0.995	0.990	72	20
**Sulfamethoxazole**	0.999	0.999	82	25
**Tamoxifen**	0.994	0.999	75	20
**Triamcinolone**	0.994	0.993	68	22
**Trimethoprim**	0.999	0.998	80	21

Notes: ICC: Instrument calibration curve; SCC: Sample calibration curve.

Mean recoveries of the selected drugs are summarised in Table 5, where recoveries higher than 60% can be observed. The highest recoveries were obtained for brilliant green, propanolol, sulfamethoxazole and trimethoprim with values above 80% and RSDs lower than 25%. Diclofenac, malachite green, sarafloxacin and tamoxifen showed recoveries between 72% and 79%, and the RSDs were not higher than 25%. The lower recoveries (60%–68%) corresponded to the quinolone drugs (difloxacin, enrofloxacin, marbofloxacin and oxolinic acid) and triamcinolone with the RSD between 22% and 27%.

Dinh *et al.* reported results for recoveries of trimethoprim and sulfamethoxazole (90% and 94%) when the spiked level was 200 ng·L^−1^ [14]. Recoveries for enrofloxacin (83%) and oxolinic acid (75%) were higher and for sarafloxacin were similar (79%). Tamtam *et al.* obtained, in general, lower recoveries with a spiked level of 100 ng·L^−1^ in river water samples using UPLC-MS/MS for trimethoprim, sulfamethoxazole, sarafloxacin and difloxacin (80%, 68%, 44% and 55%) with high RSDs (11%–29%) [17]. Recoveries for enrofloxacin were approximately 60% as in the present study. Other publications reported a range of mean recoveries between 38% to 88% for the selected drugs investigated in this study [39,47,52], lower than the values obtained in this research.

### 3.3. Monitoring the Presence of Drugs in Spanish Rivers

Of 549 water samples collected during the two sampling periods, 100 samples (18%) were positive for the presence of one or more of the selected drugs. The 13 analytes investigated were detected in at least one sample. The mean percentage of positive samples according to the type of area was 17% in urban areas, 4% in rural areas with farming activity and 2% in rural areas without farming activity. Overall, the percentage of positive samples obtained for each sampling period was similar: 19% and 17% for the first and the second, even if the number of samples collected was different, 235 (first sampling period) and 314 (second sampling period). When results for each sampling area were compared by sampling period, results were similar, particularly for those samples collected from urban areas. Sampling points located in these areas gave a higher percentage of positive samples: 16% during the first sampling period and 18% during the second sampling period. However, we noticed that even if the total percentage of positive samples detected in rural areas did not exceed 6%, during the first sampling period the percent of positive samples was double the percentage of positive samples in the second sampling period. Of all the sampling points located in urban areas, those situated after the discharge point of a WWTP (sites 3 and 13) gave the higher number of positive samples, with a range of detections between 24% and 33% of the positive samples and with a percent of detection at each site of 60% and 80% for sites 3 and 13. These results for the area selected from the Galician environment correlated with the results reported by many other authors who detected the presence of pharmaceuticals in surface water samples collected after the discharge of a WWTP [14,43,53]. Sites classified as rural areas with low farming activity were the sites with the lowest number of detections but with the highest variation between the different sampling periods. These results support the idea that contamination of the samples analysed comes from human activity.

It should be highlighted that the number of analytes detected was different in each sampling period. In the second sampling period, all the analytes selected for the study were detected in at least one sample. In the first sampling period, only four drugs were measured (diclofenac, marbofloxacin, sulfamethoxazole and trimethoprim). These results could certainly be due to a longer sampling time, as the second period was conducted over seven months while the first was conducted over only three months. Consequently, during the second sampling period, 60 more samples were collected. The fact that the second sampling period took longer may have highlighted the influence of livestock in terms of health, feeding or environmental conditions. Weather conditions were also different during the two sampling periods. Mean solar radiation was 70% higher during the second sampling period, and mean precipitation was 73% higher during the first sampling period.

**Figure 2:**
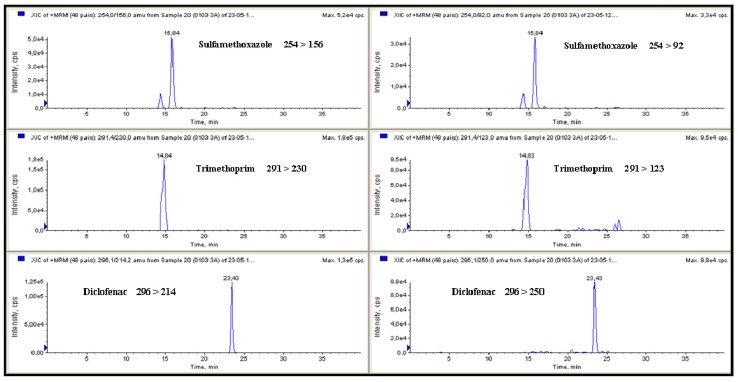
MRM chromatograms for sulfamethoxazole (37.7 ng·L^−1^), trimethoprim (64.3 ng·L^−1^) and diclofenac (31.4 ng·L^−1^) detected in galician surface water.

Analytes detected and their concentrations are summarised in Table 6 and chromatograms of a surface water sample are represented in Figure 2. If the results are assessed by the type of drugs and their frequency of detection, diclofenac was the drug with more detections in both sampling periods, with 29 (in the first) and 40 (in the second), followed by sulfamethoxazole with nine (in the first) and 33 (in the second) and by trimethoprim with seven (in the first) and 39 (in the second) also the number of detections of sulfamethoxazole and trimethoprim was in the same range for each sampling period, possibly because these drugs are usually administered together in both veterinary and human medicine as they have a synergistic effect. Their high frequency of detection could be due to their frequent use because of their low cost and their broad spectrum of activity to treat bacterial infections [54,55]. Sulfonamides such as sulfamethoxazole and trimethoprim have been detected in surface waters worldwide [56,57,58,59]. These results correlated with data reported by Huang *et al.* who suggested that sulfonamides are the most common water pollutants among various antibiotics based on information concerning their environmental fate and predicted concentrations [60]. Huang *et al.* predicted that, for example, sulfamethoxazole was most likely to be present in municipal effluent and sulfamethazine in agricultural runoff [60]. Diclofenac is a non-steroidal anti-inflammatory drug (NSAID), commonly used to treat inflammatory rheumatic diseases in humans, so its detection at sites located after the discharge of a WWTP is quite expected and, in fact, this drug was detected in 76% of the samples (*n* = 21). When the data were treated statistically, significant differences (*p* < 0.05) were found only for sulfamethoxazole and diclofenac according to sampling period. These two analytes were more frequently detected during the second sampling period when their detection was double. When a similar analysis was conducted by sampling month, no significant differences were observed for diclofenac or sulfamethoxazole, but significant differences were observed for trimethoprim. Concentration and frequency of detection of these analytes also showed significant differences (*p* < 0.05) when they were analysed according to sampling points. Site 13 was the site with the highest number of detections: 29 for diclofenac, 24 for trimethoprim, 19 for sulfamethoxazole and 10 for propanolol.

The concentrations of the drugs measured in this research were between 2.8 to 171.4 ng·L^−1^ (Table 6). Based on the therapeutic class of the drugs, antimicrobial is the group most frequently detected, with 104 detections and a mean concentration of 53.4 ng·L^−1^. The second group most frequently detected was the anti-inflammatory agent diclofenac, with 69 detections and a mean concentration of 13.6 ng·L^−1^, followed by the anti-hypertensive propanolol. The highest concentration of drugs measured during this study corresponded to sarafloxacin but this compound was detected only once, in the sampling site located downstream from the discharge of one WWTP. The detection of sarafloxacin could be due to the entrance of some animal residue into the WWTP because this fluoroquinolone is specially employed in poultry to prevent and treat infections. The drug measured at the second highest concentration (164.5 ng·L^−1^) was also a fluoroquinolone, enrofloxacin. This sample was collected at one of the sites located in a rural area with farming activity (point 7). This site is surrounded by greenery where cow droppings were found on the bank of the river, most likely due to the easy access for grazing livestock to drink water. Like sarafloxacin, the frequency of detection of enrofloxacin was low: enrofloxacin was measured in only three samples collected from three different sites (1, 7 and 13), each site classified differently. Trimethoprim was the analyte measured at the third highest concentration (110.4 ng·L^−1^). As mentioned earlier, this drug was frequently detected with a mean concentration of 13.8 ng·L^−1^ in both sampling periods. The highest observed concentration of diclofenac, the compound most frequently detected, was 45.9 ng·L^−1^, and its mean concentration was 16.6 ng·L^−1^ in both sampling periods. Jiang *et al.* published values of concentrations in the same range for sulfamethoxazole (4.86–53.24 ng·L^−1^) and trimethoprim (2.23–62.39 ng·L^−1^) in China [61]. Diclofenac was detected in the range of 2.8–46 ng·L^−1^ in 69% of the positive samples during this research. A similar frequency of detection was reported by Kasprzyk-Hordern *et al.* who detected diclofenac in rivers of the UK in the range of 1–261 ng·L^−1^ at a mean percentage frequency of 74% [51]. The greater concentration of trimethoprim (110.4 ng·L^−1^) was detected in a sample collected from a rural site surrounded by bovine livestock farms, specifically dairy cattle. Dinh *et al.* also reported concentrations for sulfamethoxazole (3.6–1,435 ng·L^−1^) and trimethoprim (8–254 ng·L^−1^) downstream of WWTPs [14]. However, if data obtained during this research are compared with concentrations of residues found in surface waters in Spain (up to 20 ng·L^−1^), the United Kingdom (up to 42 ng·L^−1^), South Korea (3.2–5.3 ng·L^−1^) and in Serbia (25 ng·L^−1^) [47,62,63,64], the concentration of the analytes measured in the Galician environment were higher.

**Table 6:** Maximum, minimum and mean concentrations of the selected drugs detected in Spanish surface water and number of time detected in the samples.

Analyte	Maximum concentration (ng·L^−1^)	Minimum concentration (ng·L^−1^)	Mean concentration (ng·L^−1^)	Number of detections
**Brilliant Green**	7.9	3.3	5.6	2
**Diclofenac**	46.0	2.8	13.6	69
**Difloxacin**	8.5	8.5	8.5	1
**Enrofloxacin**	164.5	60.0	119.9	3
**Malachite Green**	9.0	9.0	9.0	1
**Marbofloxacin**	20.1	3.6	8.8	10
**Oxolinic Acid**	39.1	39.1	39.1	1
**Propanolol**	62.6	4.2	11.5	14
**Sarafloxacin**	171.4	171.4	171.4	1
**Sulfamethoxazole**	40.1	3.0	10.7	42
**Tamoxifen**	11.7	3.5	7.5	6
**Triamcinolone**	8.4	8.4	8.4	1
**Trimethoprim**	110.4	3.5	16.1	46

Another active compound frequently detected during this research was propanolol, which is a beta blocker drug involved mainly in relaxing blood vessels and decreasing heart rate to improve blood flow and lower blood pressure. Propanolol was detected in 14 samples in the range of 4.2–62.6 ng·L^−1^, similar to the values reported by Kasprzyk-Hordern *et al.* (3–91 ng·L^−1^) in the rivers of the UK [51] and higher than the values measured by Alder *et al.* in Switzerland (7–8 ng·L^−1^) [13]. The highest concentration of propanolol found during this work (37.4 ng·L^−1^) was measured in a sample collected at a site described as a rural area. Most of the detections of this compound occurred at the sites located after the discharge of the WWTPs, most likely due to low removal during treatment at the WWTP as reported by Gros *et al.* who observed a rate of removal for this compound below 20% [65]. These results are in accordance with data published by Martín *et al.*, who detected propanolol in 100% of the samples collected downstream of discharge from four WWTPs [32]. The range of concentrations reported for this compound was between 30 to 720 ng·L^−1^, ten times higher than the concentrations measured in the River Miño. However, the area investigated was more or less than ten times less populated than the Guadalquivir area studied by Martín *et al.* [32]. As these drugs are not allowed in food-producing animals, the presence of propanolol in a sample collected from a rural area could be due to its application to animals not dedicated to food production, or the presence of this compound could also be due to a leak from the septic tanks.

The antimicrobial marbofloxacin was detected with a range in concentration from 3.6 to 20.1 ng·L^−1^, where the highest value was found at a rural site (point 1) located in the Miño River flow. The second greatest concentration was detected at a site also located in the Miño River but on its way through the city of Lugo (point 12). The rest of the analytes selected in this study were detected at a percentage less than 10%. Like sarafloxacin, oxolinic acid was detected only once (39.1 ng·L^−1^) in a sample collected at a site located near the discharge of a WWTP (point 13). Oxolinic acid had also been found in the Seine River at a similar concentration (23 ng·L^−1^) by Dinh *et al.* [14].

The presence of the triphenylmethane dyes, brilliant green and malachite green, in the Galician water is a matter for further study, even if brilliant green was detected only twice and malachite green once in concentrations that do not exceed 9.0 ng·L^−1^. As previously mentioned, these compounds are multi-organ toxics to mammals that show serious negative effects [5]. The detections of these two compounds were at the same sampling site, with two of them observed on the same day. The compounds were found at a site located near the discharge of the WWTP located at site number 13, flowing downstream of the city of Lugo. The hypothesis is that these residues could originate from a paper industry located on the upper side of the city and perhaps be swept away by the Miño River to be found in such low concentrations on the lower side or maybe due to the fish farms that are located near the city of Lugo, but this is only a hypothesis because the use of these drugs is forbidden for processing of all categories of edible fish, including fish eggs.

For each collected sample, physicochemical parameters such as nitrites, ammonium, conductivity, turbidity and pH were measured. Statistical analysis (ANOVA and a linearity test) was conducted for the concentration of the analytes and these parameters. However, no significant differences (*p* < 0.05) were found for ammonium, nitrites, conductivity, turbidity and pH. When statistical analysis was conducted with weather conditions (mean temperature (°C), mean precipitation (mm), mean humidity (%) and mean solar radiation (10 kJ/m^2^)), significant differences (*p* < 0.05) were observed for diclofenac and mean temperature. Samples that contained a measurable concentration of diclofenac were collected on days when the temperature was equal to or above 15 °C. Diclofenac was more frequently detected in samples collected in urban areas, especially after the discharge of a WWTP, but in the samples collected from rural areas, diclofenac was detected on days when the mean temperature was above 17 °C, most likely because treated animals were put out to pasture. Significant differences (*p* < 0.05) were also observed for propanolol concentration and precipitation. This analyte was generally detected on days when rainfall was not registered. The lack of detection on rainy days could have being due to a dilution effect, as most samples contained a concentration of propanolol below 10 ng·L^−1^. No significant differences (*p* < 0.05) were found for any of the drugs and mean humidity, but significant differences were observed for diclofenac concentration and mean solar radiation. Similarly to mean temperature, those positive samples for diclofenac that were collected in rural areas were collected on days with low mean solar radiation. As demonstrated by Bartels *et al.* [66], diclofenac is sensitive to solar radiation, and its concentration decreased with solar radiation. Based on these findings, diclofenac could have been liberated into the Galician environment, and its frequency of detection in this study could have been higher but solar radiation helped to decrease its concentration to undetectable levels with the method employed.

This study has demonstrated that even if Galicia is an area of Spain with high rainfall and low population density compared with other areas of Spain, the presence of active compounds such as pharmaceuticals in the aquatic environment is common, and these results were similar to results published for other areas of Spain such as Catalonia, the Spanish Mediterranean area, Valencia, Madrid and Sevilla [27,29,30,32,33,67]. Although drug concentrations were below 200 ng·L^−1^ (Table 6), toxic effects of these compounds in non-target organisms have previously been reported. Potential toxicity affected plants and soil organisms due to sulfamethoxazole [8,68] and freshwater crustaceans and fish as a result of their exposure to propanolol and diclofenac [9,69]. Based on these findings reported by other authors and results obtained for samples collected at sites located next to the collection point of a drinking water treatment plant, more than 5% of the samples collected at this site were positive for the analysed active compounds, causing significant concern.

## 4. Conclusions

The occurrence of drugs in the aquatic environment has recently become a matter of concern. This work presents a suitable HPLC-ESI-MS/MS method for the simultaneous extraction, detection and quantification of residues of 13 active substances (antimicrobials, glucocorticosteroids, non steroidal anti-inflammatories, anti-hypertensives, anti-cancer drugs and triphenylmethane dyes) in fresh water. In-house validation according to EPA guidelines makes this method suitable for government or private laboratories dedicated to investigating the presence of drugs in the aquatic environment. The method was also employed to conduct a monitoring study for the presence of the selected analytes in the Galician region with 549 water samples. All the analytes were detected and in a range of concentrations between 2.8 and 171.4 ng·L^−1^. Even if the number of sampling sites located in rural areas (with and without farming activity) was more than double the number of sampling sites located in urban areas, more positive samples were obtained in samples collected from sites located in urban areas, in particular, sites located after the discharge of a WWTP. Rural areas with farming activities also had positive samples but always at a percentage below 15% of the samples collected. The relationship between concentrations and environmental conditions, physicochemical parameters, dates and sampling points were statistically tested, and significant differences were observed only for diclofenac and temperature and solar radiation and between propanolol and precipitation. Based on these results, active compounds are also present in the Galician environment and even if farming activities are conducted in an area, the WWTPs act as a concentration point.
